# Recent Advancements in 3D Printing and Bioprinting Methods for Cardiovascular Tissue Engineering

**DOI:** 10.3390/bioengineering8100133

**Published:** 2021-09-27

**Authors:** Foteini K. Kozaniti, Despoina Nektaria Metsiou, Aikaterini E. Manara, George Athanassiou, Despina D. Deligianni

**Affiliations:** Laboratory of Biomechanics & Biomedical Engineering, Department of Mechanical Engineering & Aeronautics, University of Patras, 26504 Patras, Greece; metsiou.betty@gmail.com (D.N.M.); kmanara@upatras.gr (A.E.M.); gathan@mech.upatras.gr (G.A.); deliyian@upatras.gr (D.D.D.)

**Keywords:** cardiovascular disease, tissue engineering, 3D bioprinting, cell therapy

## Abstract

Recent decades have seen a plethora of regenerating new tissues in order to treat a multitude of cardiovascular diseases. Autografts, xenografts and bioengineered extracellular matrices have been employed in this endeavor. However, current limitations of xenografts and exogenous scaffolds to acquire sustainable cell viability, anti-inflammatory and non-cytotoxic effects with anti-thrombogenic properties underline the requirement for alternative bioengineered scaffolds. Herein, we sought to encompass the methods of biofabricated scaffolds via 3D printing and bioprinting, the biomaterials and bioinks recruited to create biomimicked tissues of cardiac valves and vascular networks. Experimental and computational designing approaches have also been included. Moreover, the in vivo applications of the latest studies on the treatment of cardiovascular diseases have been compiled and rigorously discussed.

## 1. Introduction

Cardiovascular disease (CVD) is the leading cause of morbidity and mortality globally; according to World Health Organization (WHO) more than 17.9 million people die from such causes every year—an estimated 31% of all deaths worldwide [1]. Simultaneously, the estimated healthcare cost for cardiovascular disease in Europe reaches to 169 billion € [2]. It is well established that more than 80% of CVD deaths occur in low-and middle-income countries compared to high-income countries [3,4]. Therefore, the need to reduce the economic burden is not debatable. CVD includes a wide group of complex disorders, namely peripheral arterial disease (PAD), coronary heart disease (CHD), cerebrovascular disease and rheumatic heart disease [2].

Current high-cost therapies include conventional tissue engineering, cell therapy and medical approaches [5,6]. In valve repairment, autografts are widely used by harvesting from autologous cell sources, namely parts of a patient’s body with the advent of low risk of thromboembolism and prosthetic valve infection [7]. The end stage heart failure is treated by allografting a heart from a donor, while some valve replacement surgeries employ bovine or porcine heart valves. Xenografts, though, impute other undesirable properties, including cytotoxicity and calcification [8]. Conclusively, the aforementioned grafts have their set of drawbacks, including shortage of donor organs, mechanical mismatches, anticoagulation therapy and immune rejection [9]. Synthetic valves and vascular grafts can also be implanted to treat CVD; however, the high structural durability and low rate of re-operation is outweighed by the increased risk of anticoagulation complications in patients with long life expectancy [10]. Small-diameter vascular grafts (SDVGs) constructed from synthetic polymers and decellularized matrices are promising in the field of reconstructive surgery; however, further evaluation and in vivo implementations are mandatory in order to be applied to therapeutic approaches in CVD [2].

Therefore, one showcasing solution will be the overarching focus on identifying alternative tissue treatments that preserve natural tissue without deleterious side effects.

The increased demand on recovery of damaged cardiovascular tissues in combination with the demand for low-cost but effective constructions, heralds new methods in tissue engineering [11]. Three-dimensional (3D) printing and bioprinting are the recent promising methods that successfully regenerate various organs, scaffolds and blood vessels, which can be used for replacing partly or thoroughly natural organs in the human body [12,13]. With the advent of additive manufacturing, 3D bioprinting technology employs a layer-by-layer approach which enables precise control over multiple compositions (biomaterials) and spatial distributions (cells) resulting in architectural construction accuracy [14]. Biomaterials that have been employed in 3D printing for cardiovascular tissue engineering are major natural or synthetic hydrogels, or decellularized matrices in order to mimic the dense vascular network that supports the cardiac tissue, by providing an interconnected porous network that enables cells to migrate, proliferate and receive vital nutrients and adequate oxygen supply [2,11,15,16]. This takes into consideration the survival distance limitation for cells which is no further than 100 ÷ 200 μm away from blood vessels [17]. Alginate and collagen are the most commonly used hydrogels in bioprinting following gelatin methacrylate, fibrinogen and gelatin [18]. Cell viability, proliferation and morphology after printing are crucially affected by characteristics of the selected bioink [19]. The challenge of bioink design is the improvement of printability without detracting cell viability [20]. Biomaterial-based hydrogels are capable of cell encapsulation. The major cell types that integrate the cardiac tissue are cardiomyocytes, endothelial cells, smooth muscle cells and fibroblasts. Furthermore, computational simulation with a patient’s anatomical data and features is compulsory for an integrated patient-specific bioprinted construct.

The current review article, briefly outlines the basic principles and growing applications of 3D bioprinting, highlighting key developments and in vivo implementation in the field of cardiovascular tissue engineering. More specifically, 3D-printing definitions are presented, and the 3D construct stages of manufacturing are investigated followed by the computational and experimental designing approach. In addition, different techniques of bioprinting and constitution of the inks are presented thoroughly. In closing, this review outlines recent innovative in vivo studies on 3D bioprinted applicable therapeutic approaches in CVD. 

## 2. From 3D Printing to the New Era of 3D Bioprinting 

### 2.1. Three-Dimensional Printing—Additive Manufacturing

The terms “3D printing” and “additive manufacturing” are usually confused. According to the American Society for Testing and Materials (ASTM), additive manufacturing is the process of joining materials using 3D model data layer by layer in contrast to subtractive manufacturing methodologies, such as traditional machining [21,22], whereas “3D printing” is defined as object fabrication through the deposition of a material with the help of a print head, nozzle or another printer technology [21]. The two terms are often used synonymously, especially considering they are low end in price and/or overall capability [21].

Moroni et al. tried to define “3D printing” based on the appearance of the printing process with cell-laden inks [23]. According to them, in this additive manufacturing technology a jet of binder is directed at a powder bed to define a pattern. A slice of solid material is formed after the binding of the solvent to the powder. A new layer of powder is set and by repeating this process the scaffold is build layer by layer. This definition was based on the first patent for 3D printing of Sachs et al., in which a binder solution was deposited in a powder bed according to a Computer Aided Design (CAD) model [24]. However, in the recent research of Marti et al., 3D printing is referred as the process of additive production of 3D objects, which starts from a 3D digital model [25]. Thus, the term 3D printing is still used instead of additive manufacturing for the sake of simplicity. 

#### Computational Stage—Preparation of 3D Printing

Computational methods are widely used to study tissue engineered constructs. However, the entrance of computer designing is essential in the field of tissue bioengineering due to personalized medicine. The main idea is to produce a specialized human part for each specific patient, thus contributing to a more efficient and low-cost tissue engineering [26]. The main purpose of this strategy is to create a tissue engineered scaffold with similar mechanical and biological properties concerning the defective tissue [20]. This procedure includes the following steps.

The tissue defect is digitally visualized using imaging machines, particularly CT scan (Computer Tomography scan), MRI (Magnetic Resonance Imaging) and ultrasound scan. The next step is to create a scaffold that readily supports the formation of the new tissue. The architecture of the scaffold can be meticulously designed using Computer Aided Design (CAD), a feasible way to manipulate the design parameters of tissue porosity, dimensions and biological-related properties. The scaffold can now be integrated into the 3D model of the defective tissue. Subsequently, bioink will be fabricated by assessing the proper materials, the cell types and bioactive molecules, and the location and requirements of the injured area of the patient. Eventually, using bioprinting technology the cell seeded construct can be manufactured and then placed in a cell culture or implanted directly into the patient [27]. 

Three-dimensional printing enables the precise fabrication of computationally designed scaffolds with increased accuracy, flexibility and reproducibility. These methods allow scientists to conduct low-cost parametric studies in order to create the most functional construct for the addressed medical issue. The structural design and the mechanical behavior under different conditions of the small diameter composite vascular grafts can easily be optimized by using computational methods. 

Computational methods integrate the 3D printing methods and provide the following advantages:

- more accurate techniques to model the scaffolds (e.g., image-based modelling using micro-CT), as an extra feature to reinforce the personalised medicine
- more detailed mechanobiological models to simulate different types of tissues
- more similar to in vivo conditions simulations of the scaffold’s properties and behavior under different conditions
- minimized size effect during scaffold modelling
- reduced experimental expenses (elimination of trial-and-error techniques to find the suitable scaffold)
- simultaneous estimation of the scaffold degradation and tissue regeneration in the time-dependent simulations [28,29].

A crucial issue in 3D printed tissue engineering is that the new formatted tissue may not develop adequate vascularisation for long-term survival [12]. The 3D printed construct must provide nutrients and growth factors to cells in order to proliferate [15]. However, key parameters that affect the vascularisation and need to be overcome are the shear stress from blood flow and the wall shear stress [13]. A feasible way to anticipate this challenge is through computational fluid dynamics simulations. Nowadays, personalized medicine is widely applicable and therefore three-dimensional hemodynamic simulations could contribute to the diagnosis of CVDs in order to demonstrate the needs of each patient [30]. Conclusively, flow simulations support the design and additive manufacturing techniques to produce patient-specific 3D printed biodegradable scaffolds, thus being tailored to the individual patient based on their predicted post-surgical response [31].

### 2.2. Bioprinting 

Three-dimensional bioprinting has emerged as an advanced and novel process in the field of tissue engineering and regenerative medicine. Notably, researchers’ interest in bioprinting is also evident in their efforts to define the term. According to Groll et al., Moroni et al. and Lee et al., bioprinting could be defined as the production of bio-engineered structures through computer-aided transfer processes in order to pattern and assemble living and non-living materials with a prescribed 2D or 3D organization [32,33,34]. 

Currently, the use of biomaterials in regenerative medicine and cardiovascular engineering faces challenges, including host inflammatory responses, immunogenicity, biomaterial degradation and toxicity of degradation products, that may affect the long-term function of the engineered tissue construct [35,36,37]. Therefore, the innovative biomaterial-free method of bioprinting is gaining attention in the scientific society.

Three-dimensional bioprinting has been expected to be a promising method in tissue engineering because of the ability to control precisely the geometry and the amount of biomaterials during construct fabrication [37]. More specifically, this technique can fully incorporate cells into hydrogels that satisfactorily mimic the microenvironment of the extracellular matrix (ECM) and directly print onto the targeted host location [38]. In cardiovascular engineering there are multilateral problems that need to be overcome in order to achieve an integrated 3D bioprinted model [39,40].

**Figure 1:**
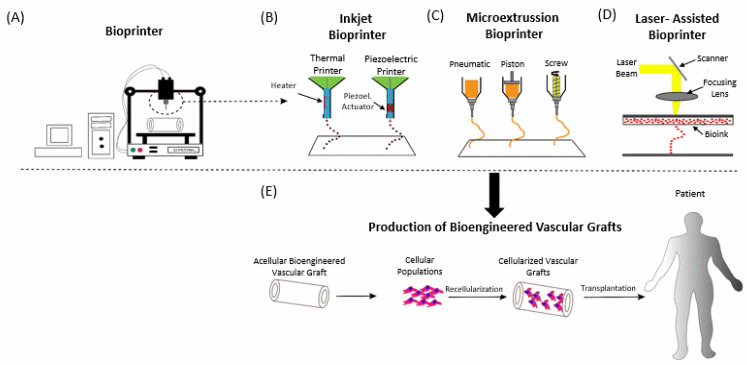
Schematic representation of bioprinters. (**A**) Development of bioengineered vascular grafts with computer assisted software. Different types of bioprinters (**B**), microextrusion bioprinter (**C**) and laser-assisted bioprinter (**D**). (**E**) Production and transplantation of bioengineered vascular grafts. Piezoel: Piezoelectric.

Cell viability and vascularization of printed tissues are key factors which determine the effectiveness of bioprinted tissues. Another impediment that needs to be overcome is the promotion of mass transfer of nutrients and oxygen into bioprinted scaffolds, including adhesion molecules and factors that induce angiogenesis [41]. The vascular tissues need substitutes with specific physical characteristics. For example, high stiffness is not favourable like in the cases of bone and cartilage tissues. On the contrary, vascular substitutes must be malleable enough to be shaped correctly in order to regenerate vessels [18]. A schematic representation of the bioprinting process and most recruited bioprinters is illustrated in Figure 1.

#### 2.2.1. Bioprinting Methods

Several studies and reviews conclude that there are three main types of bioprinting modalities: Droplet-based, extrusion-based and laser-based techniques. [14,42]. Herein, the widely applicable techniques with their set of benefits and drawbacks are briefly introduced in Table 1.

##### Droplet-Inkjet

The droplet or inkjet-based printing takes place when a bioink solution is forced under pressure and ejected as droplets through a nozzle onto an electronically controlled stage as a result of thermal or acoustic forces [14,43,44]. This heuristic technology favors the precise control of injected cells, growth factors, genes and drugs

The high resolution of the droplet-inkjet construct enables the control of the geometry and scaffold size, whilst the accuracy of cell positioning is an important advantage of the injection method. Moreover, this method is widely used in the case of blood vessels, due to the high-speed printing and the cost-effectiveness construction [14]. 

Drawbacks of this method include bioink materials with microcarriers, fragments and highly viscous hydrogels that can accumulate within the nozzle and block the flow [45]. Overall, controlling the number of cells to be encapsulated in a single droplet remains the basic challenge of this method [27].

In the Christensen et al. study, vascular-like cellular structures with horizontal and vertical bifurcations were successfully introduced using a liquid support-based inkjet, while the high post-printing fibroblast’s cell viability of printed cellular tubes was also reported [46]. Another study introduced a 3D “half-heart” scaffold with connected ventricles printed via an inkjet-based method, where mammalian cardiac cells remained viable with adequate elastic moduli and tensile strength [47]. 

##### Extrusion

In extrusion-based bioprinting, a melted polymeric filament or a cell supportive gel can be deposited. The term fused deposition modeling (FDM) is usually used to describe the first way in which a group of polymers of polycaprolactone (PCL), polyurethane (PU) and polylactic acid (PLA) can be extruded. Another way of extruding is the deposition of cell-free or cell-laden hydrogels [14]. For successful construction of vascular networks, researchers introduced and designed coaxial nozzles [48]. 

Extrusion-based bioprinting is regarded as the most practicable method since the vertical configuration is considered. Nevertheless, the induced shear stress during the printing procedure, often leads to cell death [27]. Moreover, increased shear stress can also lead to a loss of structural integrity regarding the used material, and therefore, the necessity of hydrogels which can regain the mechanical integrity is imperative [49]. 

According to Tabriz et al., the extrusion technique enables the possibility of bioprinting live human cells with an increased post bioprinting cell survival rate. In their study, alginate hydrogels were formulated with tunable mechanical properties to create straight tubular 3D hydrogel structures with diameters from 7.5 to 20 mm [50]. 

Furthermore, bioprinting of small diameter vascular grafts through coaxial extrusion possess the advantage of the simultaneous deposition of manifold materials in concentric needles [51]. Intricate multilayered 3D perfusable hollow tubes, with reported diameters 0.5 to 1.5 mm have been manufactured via coaxial nozzle [48]. In the Zhang et al. research, a coaxial nozzle system was used to print vasculature conduits with an outer diameter of 1 mm approximately, and with increased mechanical properties and bioprintability [52]. In their work, human umbilical vein smooth muscle cells (HUVSMCs), were encapsulated in sodium alginate, showed an initial low cell proliferation rate, following though an increased cell viability in prolonged in vitro cell culture.

##### Laser

In the stereolithography apparatus (SLA), a source of high-power laser solidifies a liquid resin. In a bath full of resin, the light source can produce the desired pattern [20,53]. Superior printability and cell encapsulation capacity have been reported in the biodegradable hydrogel construct in the study of Elomaa et al. [54]. Gaebel et al. [55] introduced cardiac patches for the treatment of myocardial infarction, where cardiac patch was seeded with human umbilical vein endothelial cells and human MSC, improved wound healing and functional preservation. 

The main advantage of this method is the fidelity of the achieved geometries. Complex patterns can be manufactured with high resolution, like vascular networks with wide range scales (50–250 μm) [14,56]. The contactless procedure of laser-based bioprinting prevent cells from facing mechanical stresses; thus, high cell viability is expected [53]. 

The disadvantage of the method is the necessity of photosensitive materials, and hence the selection of bioniks is limited [14,23]. Furthermore, the prerequisite of incorporating a cell type into a hydrogel restricts other possible bioinks [23]. In addition, the cost of the laser diodes is usually higher than its counterparts (nozzles). Moreover, future research could reveal the side effects in cells after the laser exposure during the manufacturing procedure [53].

#### 2.2.2. Biomaterials and Inks

##### Biomaterials

The materials that can be used in the 3D printing technique can be organic or inorganic. The inorganic inks could be categorized into metals, ceramics and glass-ceramics, and the organic ones into thermoplastic and hydrogels [20]. Titanium, cobalt-chrome, stainless steel and magnesium are some of the metallic biomaterials used. Calcium phosphates like hydroxyapatite, brushite and monetite belong in ceramics. In the glass-ceramics category, bioglass, such as silicon dioxide, calcium oxide, sodium oxide and phosphorous pentoxide, is included. As for the organic category, poly-lactic acid (PLA), polyglycolic acid (PGA) and polycaprolactone (PCL) are some of the most frequently used thermoplastics. The category of hydrogels can be divided into synthetic (poly ethylene glycol—PEG; poly vinyl alcohol—PVA; or poly acrylic acid—PAA), semi-synthetic (like derivates of hyaluronic acid, elastin and collagen) and natural (e.g., polynucleotides, polysaccharides and polypeptides) [20]. 

##### Bioinks

According to Moroni et al., material (s) and biological molecules or cells can be composed for the formulation of a bioink [23]. A myriad of current reviews summarized the recent achievements in the field of bioinks [20,42,60,61,62]. According to these studies, natural biomaterial-based bioinks, especially alginate, gelatin and fibrin, are the most cited for vascular tissue engineering applications. 

While designing a bioink, key features, namely, printability, stability, biology and rheology issues, should be seriously considered and balanced [20]. The viscosity, gelation and crosslinking capabilities are the basic characteristics to consider when selecting a bioink [19]. The deviation of the produced construct from the design depends on the bioink properties [27]. For example, an increased step in viscosity leads to an improved fidelity, but also an increased shear stress leads to cell damage and activation of misleading biophysical cues related to the ECM elasticity and pores’ characteristics [20]. 

The fidelity of the 3D manufactured construct depends on the rapidness of transition to the solid state of the bioink after the deposition. After the ejection, the decrease of gelification time improves the structure’s resolution [20]. Rheological and mechanical properties can be enhanced due to nanoparticles, with the inherent characteristic of drug delivery [63,64]. 

In Zhang et al.’s research, human umbilical vein smooth muscle cells and sodium alginate were combined, and vasculature conduits were printed through an extrusion printer, resulting in ECM formation and in increased proliferation rate [52]. Zigzag vascular tubes were fabricated through an inkjet-based bioprinter [65]. The viability of fibroblasts was at least 80% within 72 h of culture. In addition, the laser bioprinting technique improves the interplay between different types of cells and the formation of a vascular-like network [66]. 

The cell sources used in the bioprinting process could be classified into allogenic and autologous. Cardiomyocytes, human umbilical cord and embryonic stem cells were categorized in the first one, whereas adipose stem cell, skeletal stem cell, induced pluripotent stem cells (iPSCs) and bone marrow derived stem cells were put in the second category [67].

##### Maturation Methods of 3D Printed Vascular Grafts

The fabrication of vascular scaffolds is usually accompanied by post-curing methods for successful cell delivery. ECM proteins are frequently used to create a cell-supporting environment [68]. Jordahl et al. reported that extended 3D fibrillar fibronectin networks improved cell invasion and proliferation [68]. 

Efficient graft maturation favored by si RNA and poly-L-lysine (PLL) multilayers which deposited on polydopamine-coated substrates, thus a remarkable cell adhesion was noticed [69]. Low temperature plasma treatment can also be used for the treatment and maturation of polymeric scaffolds to obtain enhanced cell proliferation. More precisely, according to the research of Liu et al., nanofiber vascular scaffolds exhibited plasma treatment and the resulting hydrophilicity of these scaffolds effectively promoted vascular endothelial cell adhesion and proliferation [70]. Biocompatible photoabsorbers favor intricate scaffold maturation during the printing process. Grigoryan et al. used tartrazine, curcumin or anthocyaninc as photoabsorbers and improved the stereolithographic production of hydrogels, hence acquiring multilateral and functional vascular architectures [71]. Moreover, bioactive soft materials with enhanced biomimetic mechanical properties may result in graft maturation. Interestingly, in the Sun et al. study, magnesium ion incorporated into 3D printed polymer, where cell adhesion and proliferation were significantly promoted [72].

## 3. In Vivo Applications of 3D Bioprinting in CVD

The main aim of 3D bioprinting is to design functional tissues or parts of organs in situ for in vivo applications. The pivotal problem in terms of in vivo application is the compliance of cells and hydrogels, where cells need to precisely assemble themselves together exactly after printing, to achieve an adequate cell viability and vascularization of printed tissues. Cell–cell interaction for oxygen and nutrient interchange is mandatory to promote paracrine activity and homeostasis [73].

### 3.1. Cell Viability and Biocompatibility

Adequate cell viability is more than debatable in printed scaffolds due to high shear stresses on the cells delivered from extremely small diameter needle tips [62]. Cell viability decreases as the wall shear stress increases and the nozzle diameter of the deposition 3D bioprinting system decreases [74]. Overall, researchers should carefully select the cell density, the alginate concentration and dispensing pressure, and the coaxial nozzle size to obtain optimum cell viability on 3D bioprinted constructs [75].

Moreover, the estimation of cell viability is of paramount importance in order to decipher the interactions and stimulations between bioinks and cells, in a way that cells will satisfactorily adhere and survive [76]. Available methods for the evaluation of cell viability in 3D printed constructs are the common assays of trypan blue, release of LDH (lactate dehydrogenase), early apoptosis detection (Annexin V), Tetrazolium dye (MTT), study of DNA damage at the chromosome level (micronucleus assay) and other similar methods [77]. The optimum method to estimate cell viability, though, is fluorescent-based probes in the form of live/dead cells. Liu et al. utilized an improved in situ microscope method, where 3D constructs were split in order to investigate layer by layer the fluorescent number of cells and categorize live/dead cells [78].

Regarding in vivo studies, Bejleri et al. used bioprinted cardiac patches composed of native decellularized ECM and human cardiac progenitor cells (hCPCs). This specific combination of bioinks achieved cell viability of over approximately 75% [79]. Moreover, patches were retained on rat hearts and show vascularization over 14 days in vivo, indicating that the patches integrate well with the native myocardium inducing nutrient exchange with implanted cells.

Ong et al. suggested that in vivo implantation promoted vascularization of 3D bioprinted cardiac patches with engraftment into native rat myocardium [80]. In this study, multicellular cardiospheres consisted of human induced pluripotent stem cell derived cardiomyocytes (hiPSC-CMs), human adult ventricular cardiac fibroblasts (FBs) and human umbilical vein endothelial cells (ECs) assembled using a 3D bioprinter, and simultaneously the cell viability, in this patch, surpassed 90%.

Biocompatibility and circumvented cell cytotoxicity are mandatory in the field of 3D bioprinting materials as mentioned before. The in vivo study of Maxson et al. supports the potential use of a collagen-based bioink as an alternative for a tissue engineered heart valve implant [81]. Results of this study showed increased host cellularization potential, biocompatibility and biomechanical behavior results. The bioink was successfully printed with MSCs and showed remodeling.

### 3.2. Microarchitecture and Composition of 3D Construct Vascular Network

Three-dimensional bioprinting technology aims to combine different cell types and biomaterials heading to an enhanced cell repopulation within a 3D structure. An integrated vascular network is necessary to achieve cell viability in cardiovascular 3D printed tissues. Via that network, the influx and outflow of nutrients, metabolites and regulatory molecules are achieved. Large blood vessels ensure the flow in remote distances, whereas molecular diffusion occurs between capillaries and the surrounding tissue. In addition, the size of pores of 3D bioprinted constructs plays a major role for host cell recruitment. A pore size scaffolding >1 mm enables diffusion of nutrients until sufficient vascularization is achieved [82]. In the study of Shao et al., large scale constructs with mesoscale pore networks (100 µm to 1 mm) were successfully printed and the encapsulated vein endothelial cells were spread more efficiently compared toconstructs without mesoscale pore networks [82]. In hydrogel-based scaffolding the preferable pore size of 1–150 μm provided structural support and adequate nutrient diffusion; specifically, in the study of Zhang et al., 120–150 μm pore size resolution encouraged cells to gradually migrate into the microfibers to form a layer of confluent endothelium [83].

In the study of Maiullari et al. hydrogels and cells were printed layer by layer, thus emulating the native tissue architecture. Specifically, heterotypic human umbilical vein endothelial cells (HUVECs) and induced pluripotent cell-derived cardiomyocytes (iPSC-CMs) were transplanted hypodermically in mice and the bioprinted engineered tissue effectively merged with the host vasculature by providing enriched vascular networks [84].

Angiogenic factors play a pivotal role in the neovascularization of bioprinted cardiac tissues [85]. Notably, the tissue-engineered constructs need blood vessel development in the core. The Vascular Endothelial Growth Factor (VEGF) is used as such a regulator. VEGF regulates the vascular development and its therapeutic overexpression by the cells loaded into the construct. In this way, blood vessels sustainably grow directly into the core of the bio-engineered graft. Poldervaart et al. underlined the VEGF secretion from gelatin microparticles into the 3D constructs and the following vascularization was widely examined [85]. Further in vivo studies, regarding the effectiveness of 3D bioprinted materials, need to be implemented in order to overcome the challenge of VEGF overexpression with the intertwined side effect of vascular tumor growth (angioma) in the myocardium and other tissues [86].

### 3.3. Improved 3D Prined Grafts in Animal Models

Three-dimensional bioprinted cardiovascular grafts require robust control over a range of physical and mechanical properties that will enable bioink tailoring to a specific clinical application [62]. Overall, the greatest post-implantation challenge of 3D construct in cardiovascular tissue engineering is to maintain integrity and durability over time. Therefore, studies with animal models are necessary to improve the sustainability of 3D bioprinted cardiovascular grafts.

In the study of Melchiorri et al., 3D fabricated poly (propylene fumarate) PPF graft maintained mechanical properties, long-term mechanical support and physical parameters of graft (inner diameter and wall thickness) post six months of implantation in the venous system of the mice-selected animal model, while no thrombosis, aneurysm or stenosis were obtained [87]. In a rat animal model, 3D printed polyvinyl alcohol (PVA) mimicking 3D vascular grafts showed increased postoperative endothelialization during 30 days with significant decreased thrombogenesis [88]. Another study regarding a porcine animal model, utilized tissue engineered vascular graft (TEVG) with optimum anatomically fit and hemodynamic properties and adequate physical properties in a low-pressure venous system within one month [89].

## 4. Future Perspectives

New techniques to improve 3D bioprinting emerged due to intrinsic limitations of exogenous scaffolds or ECM-based materials [37]. Scaffold-free way of 3D bioprinting is one upcoming challenging approach to this endeavour. Tissue strands, cell sheets and spheroids, as a prefabricated block can be used for this purpose [90,91,92]. The “Kenzan” method is thought to be a pioneering method for bioprinting scaffold-free vascular grafts [93]. More precisely, spheroids are combined via micro-needles into contiguous structures. Thus, the achieved precision in a micron-level renders the method capable for tissue engineering purposes. In addition, the studies of Tseng et al. and Maina et al. introduce the magnetic 3D printing method [94,95]. Three-dimensional cellular blocks, which secrete their own ECM proteins, can be assembled with magnetic levitation. Bioinks of fibroblasts and smooth muscle cells are used for bioprinting cylindrical vessels 10 nm to 10 cm in length. Via this method, the scaffold degradation toxicity is remarkably eliminated.

Research on 3D printing and bioprinting has rapidly grown with the collaboration of various fields of expertise. Current breakthroughs in 3D bioprinting continue to broaden the spectrum of bioprinting methods and applications introducing nowadays 4D bioprinting which is expected to become the evolution of bioprinting and the next generation technology, as one more dimension of transformation over time is added [96]. In this way, dynamic 3D-patterned biological constructions could alter their microarchitecture by responding to external stimuli [97].

Merging 4D time controlled bioprinting features with innovative shape memory polymers (SMPs) paves the way to enhanced treatment in CVDs while maturation and functionalization of cells in 3D constructs alters over time [98]. Hence, the necessity of self-monitoring by regaining and maintaining their bioprinted properties over time may establish a remarkable evolution, especially in the field of personalized medicine.

## 5. Conclusions

In the realm of cardiovascular medicine, 3D bioprinting methodology leverages engineering-controlled viable biomimetic products to incorporate into clinically applicable cardiovascular grafts and tissues, heart patches, valves and other relative constructs. This review briefly summarizes the benefits and drawbacks of the 3D bioprinting method upon CVD treatment. To sum up, in order to treat a wide field of CVDs via the bioprinting method, a 3D bioprinted construct should meet the criteria of non-cytotoxicity, biodegradation, biocompatibility with preserved mechanical strength and structural integrity. Therefore, biomimicking the patient’s tissue and thoroughly incorporating into surrounding tissues and organs, thus enhancing homeostasis and construct durability and viability. In conclusion, the 3D bioprinting method still has some limitations, but has mainly tangible improvements with in vivo application for clinical translation.

## Figures and Tables

**Table 1:** Advantages and limitations of 3D bioprinting techniques for CVD treatment.

	Droplet-Inkjet	Extrusion	Laser	References
Advantages	Increased resolution and speed printing, accuracy of cell positioning, cost-effectiveness construction	Highly viscous bioinks, increased cell density, free-shape structures, most practicable method (as for the vertical configuration)	Fidelity of the achieved geometries, raised cell viability, high resolution complex patterns	[14,27,43,44,57] [48,49] [23,53,56]
Drawbacks	Low viscosity bioinks, induced mechanical forces to cells, inadequate structural integrity and cell encapsulation, use of toxic crosslinkers	Decreased resolution, cell death and degreased structural integrity due to induced shear stress	Limitation of bionics, high cost due to laser diodes, longtime of printing	[14,27,43,44,57] [48,49] [23,53,56]
Bioinks	Alginate	Alginate	Alginate, hyaluronic acid-based solutions, poly-ethylene glycol diacrylate (PEGDA) and poly-(ε-caprolactone) (PCL)	[46,47] [27,50] [54,56,58,59]
Cell type	ΝΙH3 Τ3 mouse fibroblasts, mammalian cardiac cells	Human glioma U87-MG	human umbilical vein endothelial cells (HUVEC) and human MSC (hMSC)	[46,47] [27,50] [54,55,56,58,59]
CVD application	Branched tubes with 3 mm diameter [46], Cardiac phaedo tissues (“half heart”)	Straight tubes with 7.5–20 mm diameter	Cardiac patch, tissue engineering constructs [55,56,58], branched tubes with 3 mm inner diameter	[47] [27,50] [54,59]
